# News of the Pedagogical Models in Physical Education—A Quick Review

**DOI:** 10.3390/ijerph20032586

**Published:** 2023-01-31

**Authors:** Víctor Arufe-Giráldez, Alberto Sanmiguel-Rodríguez, Oliver Ramos-Álvarez, Rubén Navarro-Patón

**Affiliations:** 1 Specific Didactics Department, Research Methods and Diagnosis in Education, University of A Coruña, 15001 A Coruña, Spain; 2 Faculty of Education, University of Camilo José Cela, 28692 Madrid, Spain; 3 Department of Education, Area of Physical and Sports Education, University of Cantabria, 39005 Santander, Spain; 4 Faculty of Teacher Training, University of Santiago de Compostela, 27001 Lugo, Spain

**Keywords:** pedagogical models, physical education, educational innovation, active methodologies

## Abstract

In the last two decades, research has proliferated in the field of pedagogical models used in school Physical Education. The growth is so high that it is necessary to do a quick review to know which models currently exist and which are emerging. The objective of this work is to collect all the models or pedagogical approaches present in the scientific literature related to school Physical Education and to make known, to the scientific and academic community, its main purposes and characteristics. A quick review of the literature found in the Web of Science and Scopus databases has been carried out using “pedagogical model” and “Physical Education” as descriptors. The results revealed a total of 19 pedagogical approaches that record scientific evidence linked to their application in the classroom. It was detected that some models or approaches were more scientifically supported than others, as is the case for Cooperative Learning, Sports Education, or Teaching Games for Understanding, while others barely registered in international scientific literature. It was concluded that researchers need to work together with Physical Education teachers to analyze the effectiveness of all these approaches. School teachers are also encouraged to vary their pedagogical approach depending on the content they are working on and the positive effects they are looking for in the psychic, motor, affective-emotional, and social domains of the students. Finally, it is proposed to researchers who promote new models or approaches a greater clarity of these to facilitate their application in the field of school Physical Education, since some difficulty has been detected in the practical application of some approaches.

## 1. Introduction

**Figure 1:**
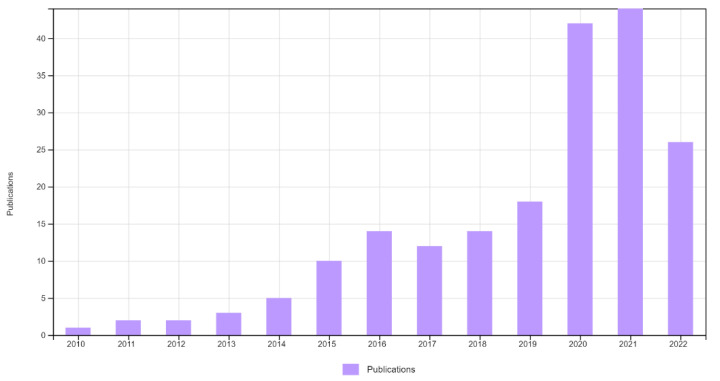
Scientific publications registered in WoS as of 22 September 2022, using as search descriptors [(Pedagogical Models AND Physical Education)] OR [(Pedagogical Model AND Physical Education)].

**Figure 2:**
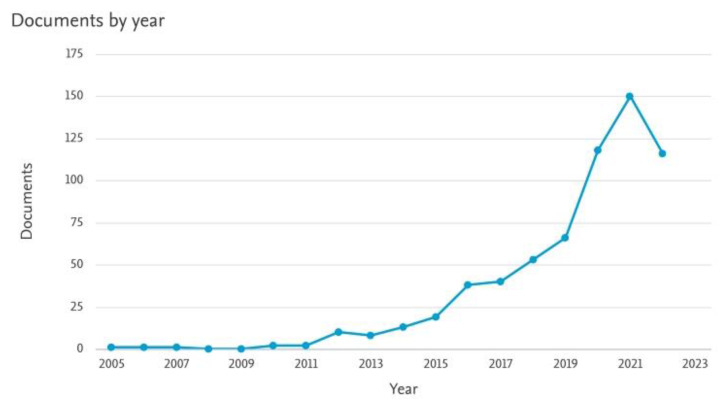
Scientific publications registered in Scopus as of 22 September 2022, using as search descriptors [(Pedagogical Models AND Physical Education)] OR [(Pedagogical Model AND Physical Education)].

In recent decades, Physical Education (PE) has experienced different changes in relation to its methodology. It has gone from instructional models to models linked to active methodologies, where the true protagonist of learning is the student body. We have the example of the use of different pedagogical models, which is supported by abundant scientific evidence that confirms improvements in learning linked to the motor, social, affective-emotional, and/or cognitive domains [1]. The number of scientific publications on pedagogical models in PE has increased in the last 20 years, registering the first publication in 2005 (Scopus) and 2010 (Web of Science) and increasing notably from the year 2020, as can be seen in Figure 1 and Figure 2, extracted from Web of Science (WoS) and Scopus, respectively.

Parallel to this increase in publications, there has also been a certain terminological confusion linked to the methodology used in the teaching–learning processes. Thus, in the scientific literature, you can find different terms that are sometimes used interchangeably, such as pedagogical models, pedagogical approaches, teaching strategies, learning strategies, teaching styles, learning techniques, and educational resources, among others. For this reason, it has been considered before presenting the current pedagogical approaches of PE teaching, to clarify this diversity of terms.

The methodology is one of the non-prescriptive curricular elements, unlike the objectives, contents, or evaluation, which are. This means that each teacher can make use of different teaching methodologies [2]. 

The didactic methodology is understood as the set of strategies, procedures, and actions organized and planned by the teaching staff, in a conscious and reflective manner, with the aim of enabling student learning and the achievement of the stated objectives [3]. Fernández Río et al. [4] distinguish four methodological levels: practical strategies that focus on a single element of the teaching–learning process (the teacher’s performance); a second level made up of teaching styles based on two elements, teacher and student production and whose main authors were Mosston and Ashworth [5]; a third level coined with the term teaching methods, where three essential elements of teacher, students, and content to teach are found; and a fourth methodological level that would be the pedagogical models. This last one could be considered the true third level. The focus of interest here is divided into four elements of the teaching–learning process: teacher, student, content, and context [4]. The pedagogical models would thus constitute the most complete level of PE didactics, including teaching styles and strategies.

In 1999, Flórez [6] defined the pedagogical model as the interrelation of the pedagogical parameters, i.e., the relations between the elements that are involved in the teaching–learning process. The successful application of a model requires a theoretical understanding of it by the teacher and correct implementation [7]. Other authors [8] point out that the models are provisional constructions, i.e., they are not absolute or determined, and may vary or disappear according to the progress of science. The models are different and alternative and do not totally displace the previous schemes, because the models are built from principles and conceptions that have already been previously addressed. They have their own distinctive practice architecture, and this is central to identifying pedagogical models [9]. Ortiz [10] concludes that there is no single, omnipotent pedagogical model capable of solving all the learning problems that students have, and that this allows for the grouping of the wide variety of typologies that have proliferated in the history of education, which have been nurtured by advances in psychology and learning theories. Metzler [7] defines the instructional model as a comprehensive and coherent plan for teaching that includes a theoretical basis, a statement of intended learning outcomes, teachers’ expertise in content knowledge, sequential and appropriate activities for their development, behavioral expectations for teachers and students, unique task structures, measures of learning outcomes, and mechanisms for measuring the faithful implementation of the model itself. Another aspect to highlight is that a pedagogical model must be didactically effective and efficient [11].

Ashley and Kirk [12] advocate using the term pedagogical model against the curriculum or instructional models used in other Models-based practice (MbP) approaches, since the word pedagogical better captures the constitutive elements of the model (i.e., curriculum, teaching, learning, and assessment).

Therefore, the concept of a pedagogical model could be defined as the planning of a teaching–learning process that, based on different psychological, social, educational, learning, and philosophical theories, takes into account why and what is going to be taught, focusing on how it will be taught, to whom it will be taught, where and when it will be taught, and how learning will be assessed. The correct application of a model must have positive effects on one or several variables of the educational process, and for it to be a model, it must have sufficient scientific evidence to support its effects on learning and have a structure that is clear, concise, and easy to apply on the part of the teaching staff. 

From this definition, some of the pedagogical models proposed by various authors would not be considered as such, instead being considered pedagogical approaches until they meet all the characteristics required to be a model. In this way, in this article, we will choose to use this last generic term to refer to both pedagogical models and possible approaches. 

It is also necessary to define the concept of PE. To do this, based on abundant scientific evidence, we propose the following definition: PE is a subject established in the educational curricula of many countries [13] that should, and can, contribute to the comprehensive development of children—improving their motor, psychic, affective-emotional, and social skills [14,15,16]—through the work of the contents established in the educational legislation of each school stage. It requires methodological and didactic principles to be adapted to the age group and stage of development of the students. It uses the body and movement as an object of knowledge and action [17,18]. It must promote adherence to the practice of physical exercise, promote a healthy lifestyle, and respond to values about the body: aesthetic, healthy, social, etc. [19]. In its practical sessions, it will make it easier for students to feel their body, know it, accept it, take care of it, develop it, and love it, as well as establish interpersonal relationships [20]. It will constitute a means for the work of motor skills, psychomotricity, physical abilities, and technical and tactical skills of different sports games and sports [21]. It promotes human capital—ethics, morals, values, psychological and psychosocial variables, and critical thinking—in the field of physical exercise, health, and nutrition [22]. Lastly, PE is an ideal subject for working on the content from other areas of knowledge in an integrated way.

Based on everything previously mentioned, the objective of this work was to collect all the proposals for models and pedagogical approaches that are linked to PE in the school environment and are currently found (with more or less scientific evidence) in the scientific literature, in order to take into account, in a single scientific document, all these approaches and to be able to follow their evolution. The description of each approach, its main purposes, and its configurator elements will be investigated. 

## 2. Materials and Methods

In order to know the current pedagogical approaches in the field of PE, a quick review has been carried out. This type of study has been chosen instead of a systematic review or meta-analysis due to the great complexity and length of the texts, which would require analyzing all the published studies on all the pedagogical models and approaches. Rapid reviews use a similar methodology to systematic reviews; however, through shortcuts used in their development, they allow answers to be achieved in less than six months and with fewer resources, which is why they are used by decision-makers both in America and Europe. They generally reach concordant answers with those obtained through a traditional systematic review [23].

**Figure 3:**
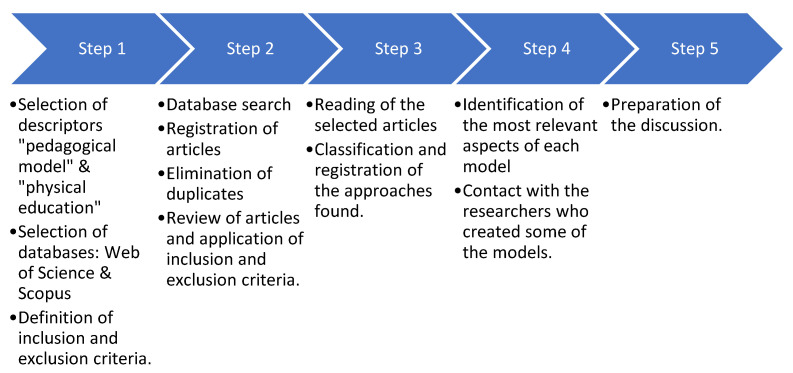
Information search process.

For the quick review, we chose to use the two databases with the most indexed scientific documents for the information search: Web of Science (WoS) and Scopus. A combination of descriptors used was [(pedagogical model AND Physical Education) OR (pedagogical models AND Physical Education) for searches within all fields and without delimiting any time filter. The research process was reflected in Figure 3. 

**Figure 4:**
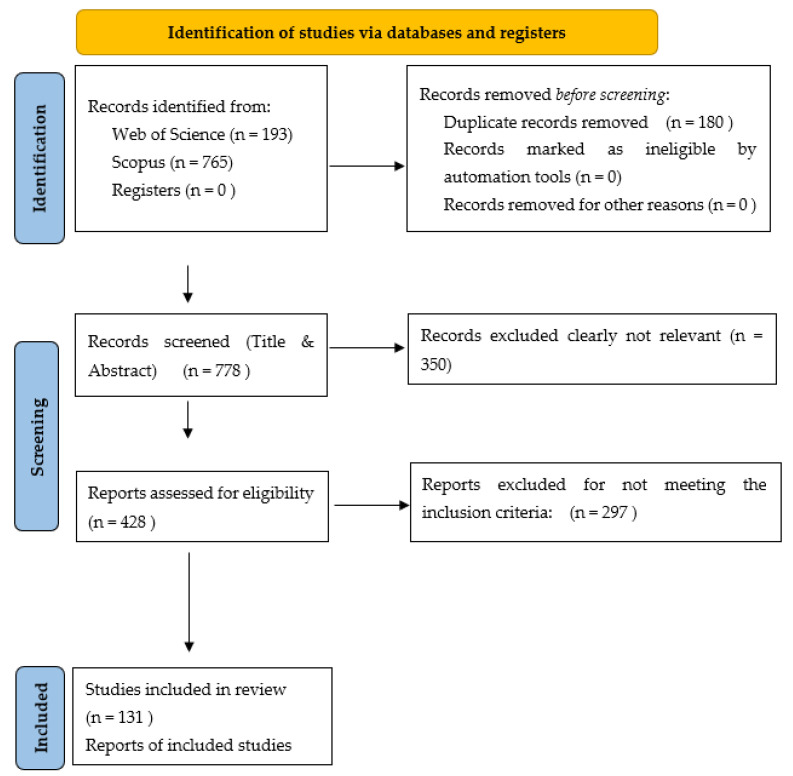
Flowchart.

The items of the PRISMA protocol [24] that the researchers considered to be most relevant for rapid review were followed (Figure 4). Ethical recommendations for educational research were complied with at all times [25].

The search was carried out in September 2022. A total of 193 documents were found in WoS and 765 documents in Scopus. Subsequently, the RefWorks^®^ bibliographic manager was used and duplicate articles were eliminated, leaving a total of 778 articles. After applying the filter of scientific articles, the inclusion and exclusion criteria recorded in Table 1, and the relevance of the works for this rapid review, a total of 131 scientific articles were selected. From these studies, the most relevant information that was of interest to this work, as determined by the opinions of the researchers participating in the search, was selected. The most important aspects of the development of each approach, its main goals, and its configuring elements were highlighted. Articles confirming the positive effects of each approach on some variables of the teaching–learning process were also selected, giving priority to systematic review, meta-analysis, or literature review articles. In cases where there was little information on a pedagogical approach, the authors of said models were personally contacted through institutional emails to delve into more bibliographic sources. 

## 3. Results and Discussion

The objective of this quick review was to analyze the different pedagogical approaches that have been addressed in the scientific literature and to establish a document that collects all the existing ones to date. Its main purpose and most outstanding characteristics, as well as some of its positive effects in the teaching–learning process of PE in the school environment, are presented. 

A total of 19 pedagogical approaches were found. In some cases, similar terms were found used by various authors to refer to the same pedagogical approach. It should be noted that it is not an objective of this review to analyze the effectiveness of each approach; thus, in this sense, it was not deepened, instead giving priority to registering the approaches found and their main characteristics to make them known to the researchers, teachers, and university students in the field of PE.

Sometimes the terms pedagogical approach and pedagogical model were used interchangeably. Thus, there is a diversity of opinions among researchers regarding what is a true pedagogical model in PE and what is not a pedagogical model. In any case, a certain level of consensus has been detected, by some researchers [26,27] in the scientific literature, when it comes to affirming that some of these pedagogical proposals are true pedagogical models, as is the case for Sport Education, Teaching Games for Understanding, and Cooperative Learning. On the other hand, Fernández-Río et al. [28] established a classification of the models based on whether or not they currently meet the formal characteristics to be a pedagogical model. Thus, on the one hand, they indicate the basic pedagogical models: Cooperative Learning, Sports Education, Comprehensive Sports Initiation (TGfU), and Personal and Social Responsibility, and on the other, the emerging pedagogical models: Adventure Education, Motor Literacy, Attitudinal Style, the Ludo-technical Model, Self-construction of materials, and Health Education. Metzler & Colquitt [7] highlight a total of 8 instructional models in the field of Physical Education: Direct Instruction, Personalized System for Instruction, Cooperative Learning, Sports Education, Peer Teaching, Inquiry Teaching, Tactical Games, and Teaching Personal and Social Responsibility.

In our case, we have found, in the scientific literature, the following 19 pedagogical approaches in the field of school PE: Cooperative Learning; Teaching Personal and Social Responsibility Model; Sports Education; Teaching Games for Understanding; Movement-Oriented Practicing Model; Service Learning; Attitudinal Style; Gamification; Outdoor Adventure Education; the Ludo-technical approach; Physical Literacy; Health-Based Physical Education; Self-Construction of Materials; Integrated Technical-Tactical Model; Flipped Classroom; Sports, Play, and Active Recreation for Kids; Exergames; Healthy Habits in Social Networks; and wearable technologies in Physical Education.

### 3.1. Cooperative Learning

Cooperative learning has the scientific solidity to be considered a pedagogical model. This approach seeks to enhance the social aspect of education. It addresses the sum of each student’s strengths and together they create something or overcome a challenge or problem. This method avoids individual Physical Education, seeking teamwork, so that students feel identified with the group and that everyone feels included and part of the group.. The sum of the parts is always a good option to promote quality education. References to cooperative learning in Physical Education have been found in the scientific literature starting from the year 2000. Prior to this date, the contribution of Johnson et al. [29] defines cooperative learning as the instructional use of small groups for students to work together to maximize their own learning and that of others. In 1966, Hill [30] stated that creative thinking arises when several people work cooperatively to solve the same problem. Currently, the application of this model requires certain non-negotiable factors for its effectiveness; thus, Johnson [29] emphasizes that cooperative efforts will be more productive if there is clearly perceived positive interdependence, considerable supportive interaction between equals, clearly perceived individual responsibility and responsibility for achieving group goals, frequent use of relevant interpersonal and small group skills, and frequent and regular group processing of current functioning to improve future group effectiveness (group self-assessment).

Polvi and Risto [31] confirmed improvements in student motivation and willingness to help others after a 9-month cooperative learning program. However, a recent literature review found that only three out of five studies showed improvements with significant differences in the motivation of the experimental group. The authors report that the duration of the intervention and the age of the participants must be taken into account in order to correctly implement cooperative learning [32]. Other researchers investigated the perceptions that students and teachers had about cooperative learning, and both groups had similar perceptions regarding the objectives of the lesson, student roles, responsibilities, communication skills, teamwork, and practice [33].

It is important to note that the term cooperation has sometimes been confused with the term collaboration, that is, cooperative learning with collaborative learning. In this last case, to fulfill the objective of the task, the joint participation of all the students is not necessary; instead, participation can be broken into parts and summed in the end. In cooperative learning, the simultaneous participation of all students must prevail in order to achieve a common goal. This confusion could interfere with the results obtained in different investigations.

Casey and Dyson [34,35,36] are two of the authors who have published the most on this pedagogical model, highlighting several publications where they explain what it consists of. Literature reviews on the effects of this model confirm that the use of Cooperative Learning in PE reported improvements in learning in the motor, cognitive, social, and affective-emotional domains, especially in the first three areas [37]. In any case, more studies are needed to investigate the benefits of long-term programs [38].

### 3.2. Teaching Personal and Social Responsibility Model (TPSR)

Developed by Hellison in 1985 [39], this pedagogical approach seeks to highlight the human capital in students, extolling their values, ethics, and morals and promoting multiple psychosocial variables. Its initial focus was aimed at students from disadvantaged backgrounds, but it has subsequently been incorporated into PE classrooms in many schools. It is a very important model where students will not only learn to know themselves better but also their projection in society and how their actions can affect the social sphere or the environment where they live [40]. It is based on the principles of responsibility, integration, teacher–student relationships, and social transfer. Some works [41] that have applied this approach in PE classes confirm improvements in motivation levels and the promotion of a healthy lifestyle. In another investigation [42], a decrease in variables related to foul play and unsportsmanlike behavior was found, thus improving the social behavior of students. The application of this approach consists of incrementally presenting tasks to students that affect personal and social development. They are basic and simple proposals that are easy to understand and whose objective is to improve the student’s attitude and behavior, emphasizing her responsibility as a member of a group. Hellison [39] established 5 levels: 1. respect for the rights and feelings of others, 2. participation and effort, 3. personal autonomy, 4. helping others and leadership, and 5. outside the sports context. The work session also has a predetermined structure, consisting of awareness, responsibility in action, group meetings and evaluation, and self-evaluation. Sports practice and PE classes are excellent means of promoting this model. It is a model that responds to the demand for active methodologies and is in continuous progress, presenting itself as an active pedagogical model [43]. 

In the scientific literature, there are several systematic review studies. The work of Sánchez-Alcaraz et al. [44], which reviewed 35 articles, found positive results in each of them—in variables such as respect, self-control, self-esteem, empathy, effort, autonomy, and cooperation—with the application of the TPSR. In another systematic review [45] where this model and the Sports Education model were analyzed, positive effects were also found on the level of respect for social conventions, respect for rules and referees, total commitment and respect for opponents, and improving fair play. The success of this model is also verified in the extracurricular environment and sports activities [46]. Finally, it is worth noting the conclusion pointed out by Pozo et al. [47] after carrying out a systematic review of 22 studies, which indicated that research with a longer intervention period is necessary; thus, carrying out a longer follow-up, quantitative methodological designs, and studies with a larger sample size is crucial.

### 3.3. Sports Education

Sports Education is another of the pedagogical, instructional, and curricular models with extensive scientific evidence. This model seeks to give students the opportunity to experience a real sports practice similar to what they can see on television or in society in general. It was created in the late 1970s by Daryl Siedentop [48], evolving later, but always providing a valuable and motivating approach through sports education to provide quality PE experiences for students from the earliest years. This model gives students great autonomy, stimulates their emotions, generates great motivation by assuming different roles within the sports field, and immerses them in sports culture, thus improving their commitment to sports. It also has a great impact on group cohesion, respect, creativity, and values. If set up correctly, it can have many benefits for students. Its most outstanding features are the combination of direct instruction, cooperative work in small groups, and peer teaching. Moreover, its goal is to help students become enthusiastic, competent, and motor-literate athletes [49]. 

This model establishes a competition system similar to that of professional teams, with preseason and season training and competition, with all students assuming different roles: physical trainers, coaches, players, judges, and referees [50]. You can even add other roles, such as psychologists, nutritionists, etc. Harvey et al. [51] contribute to this model by providing greater incidence on the ethical aspect, thus recommending four pedagogical applications within Sports Education that PE teachers, as well as practitioners and administrators of youth sports, may find useful to promote ethical development: ethical contracts, sports panels, modified games, and prizes and rewards.

Numerous studies have confirmed improvements in different variables after applying an intervention with the Sport Education model. Thus, there is an increase in the levels of motivation, literacy, and enthusiasm of Primary Education students [52] and also in Secondary Education students [53]. One of the studies analyzed [54] studied the relationship between this model and the levels of physical activity of the school population, confirming that the preseason phase is where higher levels of physical activity were recorded and that the model promoted more equitable participation, confirming similar levels of boys and girls. In a qualitative study [55] that approached a sample of 34 authors who had published numerous works on this model, it is concluded that there are certain limitations to its proliferation in the school environment due to the beliefs and values of teachers about PE and the institutional contexts and curricular policies that operate in schools; thus, further research on the model for broader curricular outcomes was recommended. Finally, the findings of a systematic review [56] that analyzed the published scientific literature with samples of children aged 6 to 18 years, where this model was applied, confirmed improvements in four learning domains: physical, social, cognitive, and affective. 

### 3.4. Teaching Games for Understanding or Comprehensive Teaching of Sports Initiation

This model is a clear example of opposition to some traditional styles that sought to focus on technical and execution aspects. It was proposed by Bunker and Thorpe in 1982 [57] under the term Teaching Games for Understanding (TGfU). The model was reviewed by Kirk and Macphail in 2002 [58], creating a more robust version since its effectiveness and application have generated much scientific discussion [27]. This model focuses on the internal logic of sports and formal and functional characteristics in order to propose games and tasks that improve the understanding of the sport, especially the principles of attack and defense, as well as the skills necessary to be able to work successfully with each sport situation. It always tries to work with situations that simulate competition, promoting modified games and the demands of each sport. It is also called the game-centered model, with some authors [59,60] proposing to enhance the teacher’s action in the intervention, and results in the application of four pedagogical principles: the selection of the type of game, game modification by representation, modification by exaggeration, and the adjustment of tactical complexity.

In the scientific literature, you can find up to five systematic reviews about it. Reviews by Oslin and Mitchell and Harvey and Jarrett [61,62] were the first to confirm that this type of pedagogy has the potential to promote change within current adult-focused cultures of youth sport and encourage participation in physical activity throughout life. Other reviews [63,64] found that most research focused on sports and sports games, and to a lesser extent on motor skills and body language. It also highlights the focus of research on game development, tactical aspects, decision-making, or technical skills. However, in general, the interventions with this model are short, recommending the authors extend its implementation. On the other hand, the latest review [65] carried out on the effects of game-based models, such as TGfU, project-based learning (PBL), and collaborative learning (CL), found improvements in physical condition, academic performance, and student enjoyment compared to traditional approaches. 

Finally, note that several researchers [66] claim that a non-linear pedagogy approach has the potential to provide researchers and physical educators with an understanding of the theoretical and practical work on TGfU in association with its pedagogical principles, thus improving the effectiveness of this model. 

### 3.5. Practice-Based Model or Movement-Oriented Practising Model (MPM)

It is a new approach that is committed to promoting the practice of physical activity inside and outside the classroom. According to Barker [66], it is based on the work of the German philosopher Peter Sloterdijk, containing a philosophical rationale and guiding principles. The articles by Barker et al. [67,68] address the full foundations of this proposal. Its main object of study is the student’s practice both in class and in their daily life. The human being needs practice to improve his abilities. The model poses an important challenge to the teacher, which is how to encourage the practice of physical activity in students without it being an instruction or obligation. How does one arouse interest in sports practice? It is necessary to make adaptations of the games and tasks for each student so that they do not fall into hatred or abandon the effort of trying to improve a skill or practice a sport. If students feel capable, they will face new challenges and practice challenges. 

It is based on the conscious practice of body movement, its purposes, the possibilities of each student, the effort in practice, and the reward it will have. It is also an approach that is closely associated with quality assessment, formative assessment, and social and personal responsibility. In words transmitted to the authors of this work personally by its author Professor Barker, 

“it is a model that is based on the idea that one can improve oneself through goal-directed practice. According to the model, practicing implies: repetition; the acceptance of standards of excellence, or verticality; uncertainty, as one can never be sure if one will achieve one’s goals; and effort. Pedagogically, teachers must: (1) provide experiences that are meaningful to individual students; (2) help students identify the knowledge to be developed; (3) help students identify cultural standards of excellence; and (4) ensure that students have enough time to practice”.

In short, the model focuses on recognizing the subjectivity and individual characteristics of each student, on providing significant challenges for each student, focusing on the contents and objectives of the practice, specifying and negotiating standards of excellence, and the last, very important aspect: to provide adequate practice time for each student, as there will be some who need more practice for the same purpose.

### 3.6. Service Learning

Service Learning (SL) has been incorporated into the educational field in recent years in many classrooms. Most of them occur within universities, with few studies that addressed their effects in Primary Education or Secondary Education within the scope of PE [69]. It is an approach that fosters values, teamwork, and social awareness [70]. It seeks to offer a service to the community after detecting a demand or a lack that may affect society. This service will be offered by the students, who through the design and programming of the work to be carried out and the intervention, will acquire important learning and also a great wealth of values. Students, thus, become the backbone of the teaching–learning process, making them participate at all times and creating moments of reflection, creativity, intervention, and experimentation. On the negative side, it entails a lot of planning time for the teacher and also a lot of coordination between the agents involved in the SL program.

### 3.7. Attitudinal Style

This approach, created by Professor Pérez Pueyo [71], seeks to focus the interest in the practice of physical activity on the attitude of the students, generating positive experiences that contribute to their greater satisfaction, encourage them to participate more, and foster a style of healthy life. It embraces principles of inclusion, cooperation, participation, formative evaluation, heterogeneity, and the progression of difficulty. In the scientific literature, there are numerous articles developed by its creator, but more references to its application at an international level are necessary [72,73,74,75]. The application of this model has also had positive effects on evaluation, confirming that the students who participated with the attitudinal style compared to traditional styles showed greater responsibility in their evaluation [76]. 

### 3.8. Gamification

Gamification has been described in the scientific literature as a technique, strategy, methodology, and even a pedagogical model [77]. Its greatest contribution is influencing the motivation of students both to participate in activities in the classroom and in tasks related to learning theoretical or practical content outside the classroom [78,79,80,81,82]. Gamification uses the elements of the game, in its mechanics and dynamics, to modify the behavior of students in a non-playful environment, such as the educational field [83]. It was born in the business field and was highly influenced by the field of psychology, being based on multiple psychological theories. In the scientific literature, there are very few experiences of gamification in the field of PE [84]. Most of the experiences belong to universities. In any case, the systematic reviews [78,84,85] on gamification do not 100% confirm its effectiveness, with their authors concluding that more studies are needed that use appropriate protocols, control groups and experimental groups, and pretest and postest measurements. A large amount of research did not take these premises into account in order to guarantee its effectiveness in learning. In addition, some authors [86] point out the importance of establishing a well-designed gamification framework to create effective gamified learning experiences, because the use of a poor framework can negatively affect student learning and motivation. In short, it is an approach that can work, since it seems to increase student motivation and physical activity levels, especially if combined with personalized learning [87]. However, it requires teacher training and knowing how to apply it, taking into account all the variables that are needed for the creation of a gamified learning environment [84,88,89]. 

### 3.9. Ludo-Technical Approach

This approach is based on teaching the technique of different sports through play, hence its ludo-technical name. It was created for the teaching of athletics, but it is currently used in the teaching of other sports. Its precursors, Valero and Conde [90], point out four outstanding phases of this model. In the global presentation and approach to challenges phase, the teacher introduces the sports discipline that will be shown in the session and carries out a practical example. Subsequently, the teacher proposes a challenge or question to the students, challenging them to solve it during practice. Then, the phase of ludo-technical proposals is incorporated, which are a series of games modified with some rules related to the technique to be learned. Then, the phase of global proposals occurs, consisting of games where the technique to be taught is practiced in its entirety. Finally, the reflection phase is an assembly where the students answer the question addressed at the beginning [91]. In short, it is about making the activities or tasks to work on the technique more attractive. The model uses direct instruction in related aspects of content selection, classroom control, task presentation, and engagement patterns [92]. To do this, games known to the students are modified, applying a technical approach, positive feedback, and training principles, such as the principle of progression of the training load, from the easy to the complex, from the specific to the global, from the concrete to the abstract, from the known to the unknown, among others, and using fun as a means. 

### 3.10. Adventure Education or Outdoor Adventure Education (OAE)

It is an approach that places adventure as a means of learning. In adventure education, students learn to take risks, assess situations, and learn more about themselves. An example of ideal activities to promote this approach are parkour, orienteering, climbing, nature outings, canoeing, etc. It is also proposed to experiment with an obstacle course (OCR) in the vicinity of the educational center if there are green areas. These are tests that work multiple muscle groups and various physical abilities. The fundamental aspects of this model are the execution of physical activity in the natural environment, experiential learning, challenges and the ability to overcome a challenge in different ways, and risk. Some authors [93] have proposed interdisciplinary work from different educational agents to enhance the benefits of this model, working together with schools and associations and establishing some recommendations proposed by experts from different countries [94]. Williams et al. [95,96] propose using the term outdoor adventure education (OAE) to refer to this model and highlight that its great strength is produced in the affective domain, promoting a positive self-concept in students, and secondarily in the cognitive and physical domains. A recent study [97], where the hybridization of this model was used together with cooperative learning and direct instruction in adolescent students, showed significantly higher intrinsic motivation and lower disruptive behaviors in the experimental group.

Finally, it is necessary to point out that the purest version of this model would not admit work within the school environment; that is, all learning experiences based on this model should be carried out in the natural environment. In any case, there are multiple researchers [98,99] who have approached the model within the school facilities and also verify multiple benefits for students.

### 3.11. Motor Literacy or Physical Literacy (PL)

It is an approach that emphasizes the importance of promoting and working on motor skills as a source of knowledge to facilitate decision-making in students when they interact with the environment, thus promoting adherence to the practice of physical activity [50]. Capel and Whitehead [100] describe it as an acquired disposition by individuals that encompasses the motivation, confidence, physical competence, knowledge, and understanding to maintain physical activity throughout life. David Kirk [101] points out that physical literacy could constitute a new model based on an existentialist philosophical perspective. Finally, it should be noted that various works [50,100,101,102,103] recommend the hybridization of the Sport Education and Physical Literacy model, affirming that it can be successful and achieve greater benefits for students, as both proposals complement each other very well. 

### 3.12. Education for Health or Health-Based Physical Education (HBPE)

The importance that education has for the health of the population has been confirmed by multiple researchers [104] who have insisted on the need to address Physical Education that enhances the health of students rather than a reductionist version or even a version that is potentially harmful to the health of students [105]. In this way, a pedagogical proposal for health education, whose main purpose is the promotion of healthy habits in students, also arises. Haerens et al. [106] based on the contributions of Jewett et al. [107] and Metzler [7], propose this approach. His proposal presents the need for students to positively value a physically active life, knowing how to identify the appropriate physical activities at all times to improve their health and well-being throughout their current and future lives. The model affects the field of self-realization, social reconstruction, and affective domains. 

### 3.13. Self-Construction of Materials

This pedagogical approach is widely used in Spain by Professor Antonio Méndez [108], with the publication of a book dealing with the self-construction of materials in Physical Education. Later, he published multiple articles, sometimes with didactic proposals [109,110] and other quantitative and qualitative research [111,112,113,114]. This approach is created from a constructionist learning perspective [115], where students are the ones who build their own material—mainly from recycled material—experiment with it, and analyze its operation, thus improving their knowledge. During this process, students begin a conversation with other peers, favoring self-directed learning, constructivism, and constructionism [116]. The self-construction of materials fulfills a double function, alleviating the material deficit that the PE department of educational centers usually has and promoting different domains of human development, including motor, cognitive and affective-social, but also artistic domains [111]. Some authors have approached this approach and obtained numerous benefits for students in variables of enjoyment [117], motivation, and values, among others [118]. 

### 3.14. Integrated Technical-Tactical Model (MIT-T)

This pedagogical proposal was created by López and Castejón, with a first publication in 1997 [119]. Its main purpose is to promote the development of technical and tactical thinking from individual and group actions. Favoring, in students, the understanding of the game structures of different sports in their technical and tactical action and their strategic principles [120] is the goal. The MIT-T is inspired by the TGfU and seeks to promote contextualized, comprehensive, and as meaningful learning as possible. No scientific evidence of the application of this proposal in the international arena has been found, except for the review of the model published by López and Avelar [121], where they emphasize that the model is based on the constructivist perspective of teaching–learning, particularly characterized by teaching both techniques and tactics in a contextualized way and applying learning in simplified game situations that allow for the contextualization of learning in more complex scenarios.

### 3.15. Flipped Classroom

The Flipped Classroom has been considered by various authors as a pedagogical model [122,123]. This pedagogical approach is practically based on a teaching–learning model where the teacher designs lessons using RICT (relationship, information, and communication technologies) that are attractive to the students so that they can view them at home. Once they are viewed later in class, they practice, discuss, and perform tasks linked to those contents shown through RICT. In the field of PE, various experiences have been recorded with this model, obtaining positive results in relation to the involvement and motivation of students and also of families [124,125]. A systematic review [126] confirmed that the Flipped Classroom is more effective than other methodologies, in terms of learning achievement, in secondary and higher education and could be more beneficial than other methodologies in variables such as motivation, self-efficacy, or commitment. Its authors also emphasize that more research is needed to verify its effectiveness at all educational stages. 

### 3.16. Sports, Play, and Active Recreation for Kids (SPARK)

This educational program was created in 1989 by the San Diego State University Research Foundation as a curricular program to promote the practice of physical activity both in the school environment and outside of school. SPARK programs were designed in response to a societal need to combat low levels of physical activity and fitness in children [127]. It seeks to improve the health of students, their socialization, and their enjoyment of practicing sports. It has much scientific evidence, including the improvements obtained in academic performance [128], levels of physical activity [129,130], motor skills [131,132], and the physical condition of students with disabilities [133]. The model is composed of a curriculum for physical education of sports, games, and active recreation for children. It is a model for evidence-based, research-based, health-related physical education programs in the United States [134]. Initially, it was focused on the infant stage and later it was extended to primary and secondary school.

### 3.17. Active Video Games or Exergames

Active video games have also been the subject of research by the scientific community and have been applied in school settings with positive results. This pedagogical approach favors the practice of physical activity in students [135,136,137], the improvement of their motor skills [138], and also the motivation of students [139]. However, some authors [139] question whether exergaming is a sustainable way to motivate children. Other works [140] found more improvements in aerobic physical performance than with the application of the SPARK model. Highlight the reflection made by Cheng [141] on the possibility of incorporating the practice of exergames into the PE curriculum as a result of the scientific literature compiled to date, a reflection also shared by other authors [142] who have carried out a review on the subject.

### 3.18. Promotion of Healthy Habits in Social Networks

Social Networks are also presented as a means of work in the educational field. There are multiple studies that have analyzed its effects on different variables linked to the teaching–learning process [143,144,145]. In contrast to the benefits, the ethics of research and the use of social networks in the field of EP have also been questioned [146]. However, various authors [147] challenge the educational community to incorporate its use in the field of PE, even working on hybridization with the physical literacy model or with the Sport Education model [145] and obtaining positive results, such as greater participation of families and involvement of students. They can thus be integrated into PE and health pedagogy. Other authors [148] have investigated social EP, which is linked to mobile learning where students use electronic devices to improve their learning, the mobile phone being one of them and being in full development.

### 3.19. Wearable Technologies in Physical Education or Wearable Technologies

In today’s society, numerous fields of knowledge, such as engineering, nursing, medicine, psychology, and PE itself, have a significant interest in wearable technology for health management [149]. The future of PE may lie in this new pedagogical approach that we present here, using the benefits that wearable technologies offer us. Some review works [150] confirmed the benefits of the use of digital media in PE in motivation or improvement of sport-specific motor skills and abilities but also confirmed a certain lack of knowledge of teachers in the use of these devices. These authors confirm that few studies specifically addressed learning through digital media. More specifically in the field of wearable technologies, such as activity bracelets or watches that measure steps, rhythm, caloric record, etc. Some studies [151] have found improvements in the motivation of students to practice physical activity. The hybridization of exergames and wearable technologies was also successful in some studies [152] with the researchers confirming improvements in PE classes in the group of students who used the exergame Running Othello 2 (RO2) together with a bracelet and smartphone, the players became more engaged and their heart rates increased. Several authors [153] have used with positive results the application of technological systems in PE classes that help the teacher to observe the amount and state of movement of the students in real-time, to improve the quality of teaching. Even a team of researchers [154] has proposed the use of the so-called WST model, a model that is created to help teachers understand each student’s timely exercise load, adjust training activities, and issue feedback as an early warning if they are working above or below the desired physical exercise load. This is important, and this is also confirmed by Dong et al. [155] pointing out that it is necessary to scientifically and accurately judge the exercise load of students and guarantee the safety of their exercise in Physical Education classes. Teachers can use this technology to know the physical exercise data of the students. 

### 3.20. Pedagogical Approaches That Were Not Considered 

For this article, other pedagogical proposals have not been considered, such as formative and shared evaluation, since it is considered that, more than a pedagogical model, it is an evaluation model facilitating dialogue between teacher and student and betting on a continuous process where students participate in said evaluation [156,157].

Self-regulation of learning is also not addressed as some authors refer to it as a distinctive approach to academic learning and instruction based on self-regulation theories [158] and not so much a pedagogical model. Neither any of the considered teaching styles, such as direct instruction, guided discovery, and problem-solving, nor any of the 11 styles proposed by Mosston and Ashworth [5] for being styles and not pedagogical models.

Another approach that we have not considered but that really is a transversal pedagogical model that can coexist with other models or approaches is the universal design for learning. This is defined by CAST [159] as “a framework to improve and optimize teaching and learning for all people based on scientific knowledge about how humans learn.” There is still little published scientific literature on this model in the field of Physical Education, but we can find some articles that address how to include it in Physical Education classes [160,161,162].

### 3.21. Other Observations on Pedagogical Approaches and Models

Despite being an interesting proposal, the classification of pedagogical models in PE was created by Fernández Río et al. [163] in which two types of pedagogical models are proposed: basic or consolidated and emerging. We believe that calling an emerging model a pedagogical approach or teaching/learning strategy that does not yet meet the characteristics to be a model can lead to confusion. Instead, we consider using the generic term pedagogical approach. This term is more in line with reality and avoids spreading the confusion generated among PE teachers and the academic community as to whether an emerging model is really a pedagogical model. The name itself seems to confirm that it is a model, that it has already been born or has been created, and is beginning to increase its presence when it really is not a model. This fact should not be perceived with a negative connotation, but rather tries to convey that the proposal has the appearance of a model but it really is not at present and maybe in the future. It should also be noted that a pedagogical approach that has little scientific evidence does not mean that it does not work as a model, but rather that more evidence is needed to consolidate its positive effects. 

Finally, it is important to highlight the findings of Fjellner et al. [164] who carried out a scoping review on pedagogical models and how PE teachers assume their implementation in the classroom. The authors conclude the existence, on the one hand, of the researchers who investigate the effects of these programs and, on the other hand, of PE teachers who are in the classrooms, and who are somewhat reluctant to use models or perceive disempowerment. In their work on PE pedagogical models, teachers positioned themselves as: (1) resistant to the use of models; (2) unable to use models correctly; (3) mechanical model players; (4) struggling model implementers; (5) needing models to change their ordinary practices; (6) able to use models correctly with support; (7) model adapters, and (8) researchers’ collaborators in model implementation. This may be an indicator that pedagogical models may have more presence in research and not so much in ordinary PE classrooms, being more necessary the connection between researchers and Physical Education teachers more in order to be able to transmit this scientific evidence to obtain a greater use of its benefits in Physical Education classes.

Other authors [103] have recently shown their concern in this area, highlighting up to three types of collaborations between researchers and PE teachers and concluding the need to overcome the belief that the researcher knows more than the PE teacher, giving teachers tools to be a researcher.

Finally, as limitations of this work, the reader is informed that, despite having consulted two important databases, such as WoS and Scopus, and has selected a series of articles to support the results and discussion of this work, there is the possibility of the existence of some other pedagogical approach not included here, as well as other possible positive or negative effects of the implementation of models in the PE classroom. Due to the large amount of information that brings together this rapid review work, it was not possible to analyze all the scientific publications found in the databases, producing a small bias as the researchers selected the articles that, in their opinion, could contribute more to the writing of this text. In any case, the authors declare that they have no conflict of interest. 

## 4. Conclusions

The objective of this work was to collect, in a single document, the pedagogical approaches currently present in school PE and to analyze their main configuring elements and purposes. A total of 19 possible pedagogical approaches have been found, with their corresponding strengths. Many of the works emphasize the importance of the hybridization of models to achieve greater improvements in the affective, physical, social, and psychic domains of children. A greater amount of international scientific evidence has been observed in the application of certain pedagogical approaches, such as sports education, the Teaching Games for Understanding (TGfU) model, or cooperative learning. Other approaches, however, have little international scientific support, such is the case for the Attitudinal Style, Ludo-technical, and MIT-T approaches, among others, thus inviting researchers to apply them in their respective countries. Pedagogical approaches that have proliferated in recent years and that may, in the future, be consolidated, such as wearable technology or the use of RRSS to enhance PE work, were also detected. After this quick review, and based on the results obtained, three important takeaways were concluded. First, inviting PE teachers to experiment with new teaching approaches for teaching the content they teach is needed. Secondly, the scientific community is invited to work collaboratively with PE teachers in researching the effectiveness of the different pedagogical models. Thirdly, the configuration bases and the application and implementation of some approaches—which, due to their structural complexity, are not reaching the field of school PE well, and consequently, lack a solid scientific endorsement—require clarification. Through working on these three points, it will be possible to better analyze the effectiveness of each pedagogical approach.

## Figures and Tables

**Table 1:** Inclusion and exclusion criteria.

Inclusion Criteria	Exclusion Criteria
1.a. Scientific papers were published in the form of a peer-reviewed scientific article	2.a. Publications that did not have access to at least the abstract.
1.b. Research of any kind (experimental, reviews, descriptive, etc.).	2.b. The pedagogical model and Physical Education were not part of the study.
1.c. The research was on School physical education and the pedagogical model.	2.c. Documents were not published in the form of a peer-reviewed scientific article, for example: theses, conferences, editorials, opinion articles, etc.
1.d. Publications indexed in Web of Science or Scopus, provided they were in the English language, at least in their title, abstract, and keywords.	2.d. Duplicate items
	2.e. Made reference to Physical Education at university, in Vocational Training, or the extracurricular field.

## Data Availability

Not applicable.
